# Simple and sensitive electrochemical sensing of amethopterin by using carbon nanobowl/cyclodextrin electrode

**DOI:** 10.1016/j.heliyon.2024.e31060

**Published:** 2024-05-10

**Authors:** Jian Wang, Xiuzhi Xu, Zhulai Li, Bin Qiu

**Affiliations:** a Pharmaceutical Chemistry Department, School of Pharmacy, Fujian Key Laboratory of Drug Target Discovery and Structural and Functional Research, Fujian Medical University, Fuzhou, 350122, PR China; b Ministry of Education Key Laboratory for Analytical Science of Food Safety and Biology (Fuzhou University), Fujian Provincial Key Laboratory of Analysis and Detection for Food Safety, Eel Farming and Processing, Fuzhou, Fujian, 350108, PR China

**Keywords:** Amethopterin, Carbon nanobowl, Cyclodextrin, Electrochemical sensor, Modified electrode, Antineoplastic drug

## Abstract

Resulted from the severe side effects, the development of inexpensive, simple and sensitive method for amethopterin (ATP, an antineoplastic drug) is very important but it still remains a challenge. In this work, low cost nanohybrid composed of carbon nanobowl (CNB) and β-cyclodextrins (β-CD) (CNB-CD) was prepared with a simple autopolymerization way and applied as electrode material to develop a novel electrochemical sensor of ATP. Scanning-/transmission-electron microscopy, Fourier transform infrared spectrum, photographic image and electrochemical technologies were utilized to characterize morphologies and structure of the as-prepared CNB and CNB-CD materials. On the basic of the coordination advantages from CNB (prominent electrical property and surface area) and β-CD (superior molecule-recognition and solubility capabilities), the CNB-CD nanohybrid modified electrode exhibits superior sensing performances toward ATP, and a low detection limit of 0.002 μM coupled with larger linearity of 0.005–12.0 μM are obtained. In addition, the as-prepared sensor offers desirable repeatability, stability, selectivity and practical application property, confirming that this proposal may have important applications in the determination of ATP.

## 1 Introduction

As a widely used antineoplastic drug, amethopterin (ATP) is generally used for the treatment of many tumours, such as breast, oral tumor and lymphoma [1,2]; meanwhile, it's also utilized widely as one effective drug for several autoimmune diseases including lupus, psoriasis, and rheumatoid arthritis [3,4]. Nevertheless, high-dose ATP can induce many side-effect, typically such as ulcerative stomatitis, liver damage, and even life threatening [5,6]. In consequence, designing effective method to monitor ATP degree in the human blood is considerable necessary to know the drug dose for achieving best treatment effect with smallest toxicity.

So far,several typical techniques have been developed to detect ATP, including mainly capillary electrophoresis, fluorimetry, ultra violet-visible spectrometer, high pressure-liquid chromatography, and mass spectrometry [[7], [8], [9]], but which generally require complex pretreatment and operation processes, overmuch organic solvent or high cost, restricting their practical application [10,11]. Lately, electrochemical method has attached some attentions to monitor ATP degree attributed to its dominant advantages in simplicity, cost and sensitivity [[12], [13], [14]]. For example, Jandaghi et al. [11] prepared nano Ce–ZnO flower to construct a voltammetric sensor for ATP detection, this work revealed Ce–ZnO electrode possesses a low limit of detection (LOD, 6.3 nM); while Fathi and his colleagues [15] fabricated a CuCr_2_O_4_/CuO electrode to detect ATP, achieving a linearity of 0.1–300.0 μM and a LOD of 2.5 nM. In another interesting works, Asadian et al. [16] synthesized a graphene/CNTs hybrids and successfully utilized it to detect ATP electrochemically; Tong and Chen's group [17] prepared poly (L-cysteine)/g-C3N4 modified electrode to detect ATP, and the obtained LOD value is low to 6.0 nM. Although these methods are highly effective and have great applications in the real detection of ATP, developing simple and sensitive electrochemical sensor for ATP still remains a challenge.

As is well-known, many carbon-based nanomaterials have been utilized widely in numerous research fields owing to their dominant properties [[18], [19], [20], [21]]. Carbon hollow nanospheres (CHP), a typical three-dimensional carbon nanostructure, could provide various attractive merits, e.g., high conductivity, surface area and active sites, received intense concerns in constructing electrochemical sensors [10,22,23]. And many interesting CHP-based electrochemical sensors have been designed, showing important real applications [[24], [25], [26]]. Nevertheless, what's easily neglected is the full spherical structure of CHP restrains the related availability factor in its internal-surface, thus decreasing its actual performance [[27], [28], [29]]. Carbon nanobowls (CNB), a new star carbon nanostructure with unique open bowl-like structure proposed in 2019, presently has been concerned in several fields (e.g., energy storage, drug delivery, pollutant catching and nanoreactor) [[30], [31], [32]]. This bowl-like structure enables CNB with a larger surface area and availability factor about the internal surface, inducing more superior performance [[33], [34], [35]], enabling CNB to become a superior candidate in designing an electrochemical sensor for ATP analysis. However, the unmodified pure CNB are usually insoluble in water, resulting in the severe restriction of its application.

**Scheme 1:**
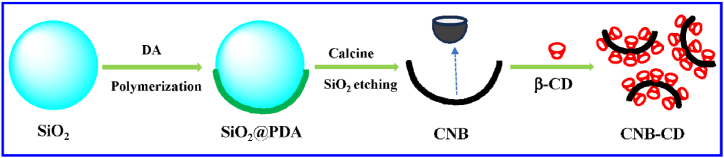
The schematic illustration for preparing CNB-CD nanohybrid.

As a simple oligosaccharide, β-cyclodextrins (β-CD) is considerable cheap and can be used to improve the water-solubility and dispersibility of the β-CD-based nanohybrids as the solubilizer [[36], [37], [38], [39], [40]]; meanwhile, the β-CD molecule exhibits excellent molecule recognition and accumulation capability [[41], [42], [43], [44]]. Thus in this work, the nanohybrids composed of CNB and β-CD (CNB-CD) was prepared with a simple way for the first time (Scheme 1) and dedicated to fabricate a low-cost, simple, and highly sensitive electrochemical sensing platform of ATP. Owing to the collaborative advantages of CNB and β-CD, the CNB-CD hybrid modified electrode shows superior analytical performances towards ATP via optimizing various conditions, thus offering a novel electrochemical strategy for ATP detection with low-cost, simpleness and high sensitivity.

## 2 Experimental section

### 2.1 Reagents and instruments

CD was obtained from Shandong Binzhou Zhiyuan Co. Ltd. (China). ATP, SiO_2_ (1.0 kg/8$), hydrofluoric acid (HF, 40 %) and dopamine hydrochloride (DA, 1.0 g/12$) were purchased from Zhenjiang Dewei Co. Ltd. (China). Uric acid (UA), folinic acid (FA), ascorbic acid (AA), pyridoxine (Py), dopamine (Dp), tetrahydofolic acid (TFA) and 5-methyltetrahydrofolate (MHF) were purchased from Aladdin (China). The utilized electrolyte is phosphate buffer solutions (PBS, pH 6.5) which was achieved with Na_2_HPO_4_, KCl and NaH_2_PO_4_. The electroanalytical experiments were carried out at a CHI 660E Electrochemical Workstation and the glassy carbon electrode (GCE) was introduced as working-electrode.

### 2.2 Preparation of CNB-CD and modified electrodes

Before preparing CNB-CD, the synthesis of CNB was performed via a simple autopolymerization method: SiO_2_ powder of 300 mg and DA of 300 mg were mixed in 100.0 mL Tris-buffer (pH 8.5) to make DA autopolymerization on the surface of SiO_2_. After centrifugation and drying, the product SiO_2_@polydopamine (SiO_2_@PDA) was obtained. Next, via calcining SiO_2_@PDA at 800 °C in N_2_ atmosphere and etching the SiO_2_ template by HF, CNB was collected after washing and drying.

Next, the CNB-CD nanohybrid was prepared via an easy ultrasonication way. Specifically, CNB of 10.0 mg and β-CD of 10.0 mg were mixed and consecutively ultrasonicated for ∼20 min in 20.0 mL water, thus a homogeneous and black dispersion of CNB-CD (1.0 mg mL^−1^) was obtained. For preparing CNB-CD/GCE, the 8.0 μL CNB-CD dispersion was coated on GCE and dried well.

## 3 Results and discussion

### 3.1 Characterization for the as-synthesized CNB-CD hybrid

**Fig. 1:**
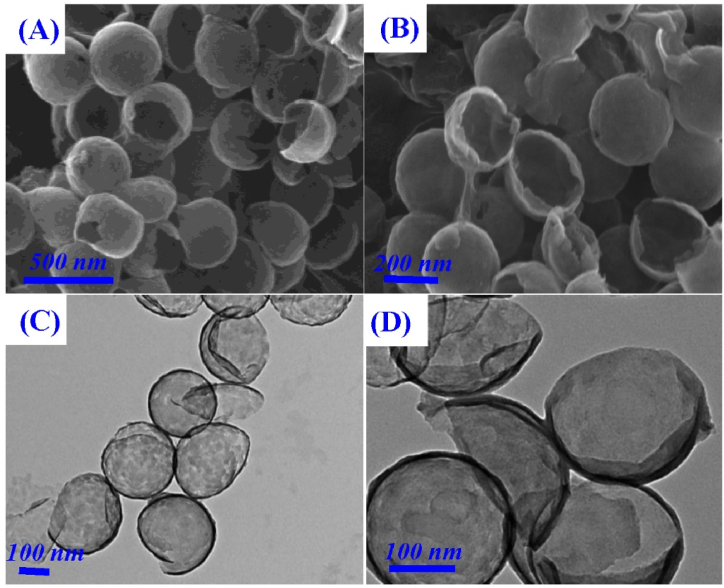
The SEM images of (A) CNB and (B) CNB-CD; the TEM images of (C) CNB and (D)CNB-CD.

**Fig. 2:**
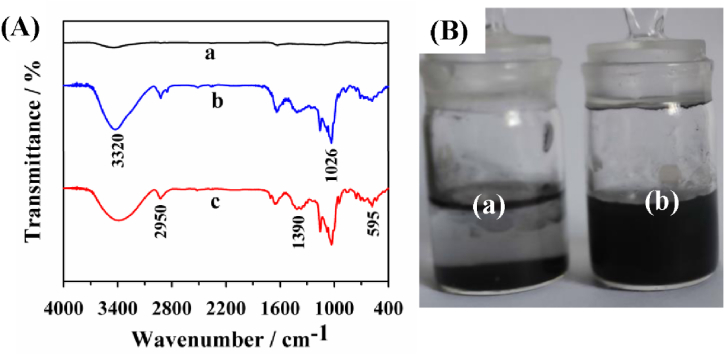
(A) The FT-IR spectrums of (a) CNB, (b) β-CD and (c) CNB-CD; (B) the photographic images of 1.0 mg mL^−1^ (a) CNB and (b) CNB-CD dispersions in water after ten days of storage.

The scanning electron microscope (SEM) and transmission electron microscopy (TEM) were firstly adopted to study the morphologies of CNB and CNB-CD. As displayed in Fig. 1A and C, a bowl-like, open structure with ∼320 nm diameter can be observed for CNB, indicating CNB was prepared successfully. This bowl-shaped structure is beneficial to enhance the utilization ratio of the internal surface and provide abundant defect structure, inducing prominent sensing ability. When β-CD molecules were attached on the CNB surface, the morphology of CNB shows no obvious changes (Fig. 1B and D), indicating the β-CD molecules would not destroy the bowl-like structure of CNB. Meanwhile, the β-CD attachment to the CNB surface was also studied via Fourier transform infrared spectrum (FT-IR) spectra. As displayed in Fig. 2A, there is nearly no obvious peak observed for pure CNB, while β-CD exhibits several typical peaks at 595, 1026, 1390 2950 and 3320 cm^−1^, which are refer respectively to the ring vibration, coupled C–O/C–C stretching, C–H/O–H bending vibration, CH_2_ and the O–H stretching vibration [10,45]. As for the as-prepared CNB-CD nanohybrid, most of β-CD peaks could be observed, demonstrating the successful formation of CNB-CD composite. In addition, Fig. 2B displayed photographic images of the pure CNB and the as-prepared CNB-CD dispersion, the results showed that the pure CNB nanoparticles tend to aggregate with each other resulted from the weak solubility, but the CNB-CD dispersion presents well-dispersed and homogeneous solution after ten days of storage.

**Fig. 3:**
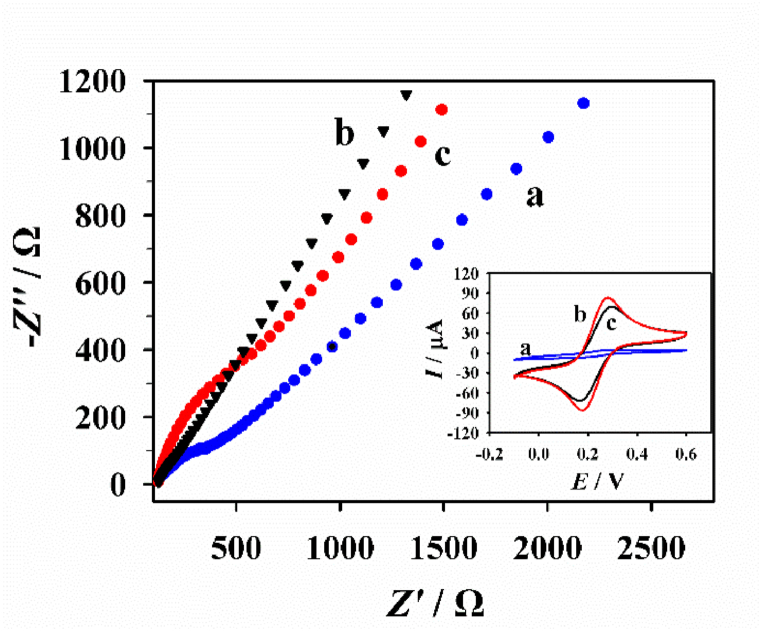
The EIS plots of bare GCE (a), CNB/GCE (b) and CNB-CD/GCE (c) in [Fe(CN)_6_]^3-/4-^ solution. The inset is the related CV responses of various electrodes.

Next, the electron-transfer-resistance (*R*_ct_) properties of CNB and CNB-CD modified electrode were compared by electrochemical impedance spectroscopy (EIS) which was evaluated in the [Fe(CN)_6_]^3-/4-^ solution of 5.0 mM (Fig. 3). Based on the parallel circuit, it's found *R*_ct_ value at CNB/GCE is extremely small (∼50 Ω) compared with that at bare GCE (∼350 Ω), demonstrating CNB possesses excellent electrical property. Resulted from the β-CD attachment to the CNB surface, a weak increase of the *R*_ct_ value (∼270 Ω) can be observed at CNB-CD/GCE. Cyclic voltammetry (CV) behaviors of the electrodes in the [Fe(CN)_6_]^3-/4-^ solution were studied also and the formed results are similar to those obtained through EIS.

### 3.2 Electrochemical detection of ATP

**Fig. 4:**
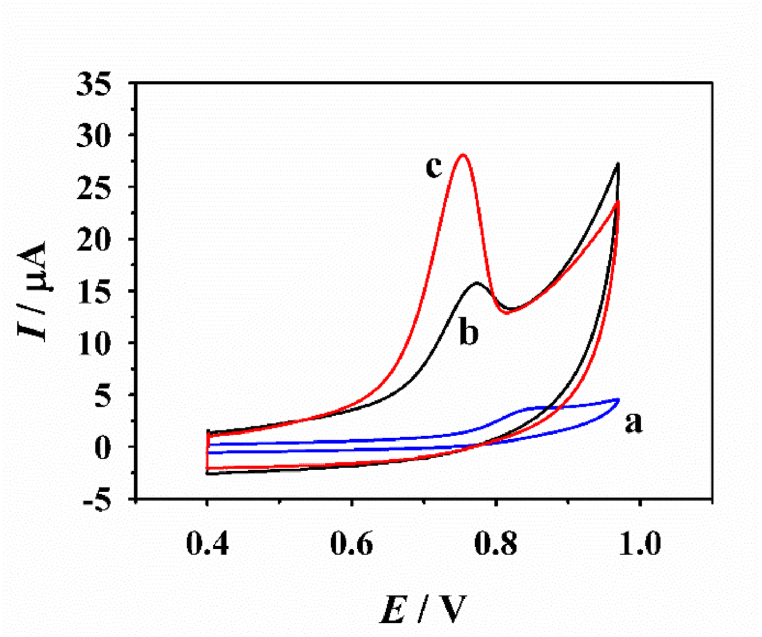
The CV responses of the bare GCE (a), CNB/GCE (b), and CNB-CD/GCE (c) to 50.0 μM ATP.

**Scheme 2:**
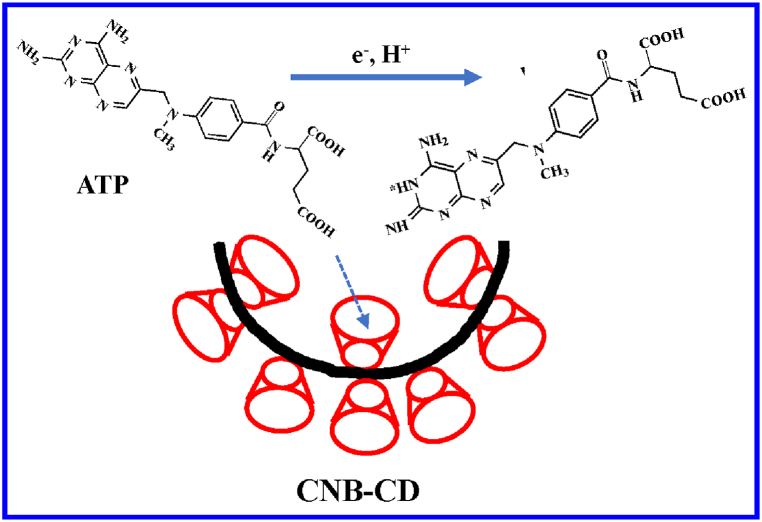
The electrochemical-oxidation mechanism of ATP at CNB-CD/GCE.

Firstly, the feasibility for the determination of ATP based on the as-prepared CNB-CD/GCE was verified through cyclic voltammetry (CV), and Fig. 4 displayed the CV responses of 50.0 μM ATP at various electrodes. It was found the oxidation of ATP at bare GCE exhibits a weak peak current at ∼0.82 V, while this peak current increases spectacularly and the peak potential shifted negatively to 0.76 V at CNB/GCE due to the prominent electrical property and larger surface area from the pure CNB. As for the oxidation of ATP at CNB-CD/GCE, the peak current increases still substantially which is ∼3.2 times higher than that at CNB/GCE attribute to the high recognition capability and hydrophilic cavity of the β-CD structure, and Scheme 2 displayed the related electrochemical oxidation mechanism of ATP: the β-CD molecules not only provide ideal host to accumulation ATP molecules but also enhance the dispersibility of CNB due to its hydrophilic functionality, and CNB offers prominent electrical property and large surface area.

**Fig. 5:**
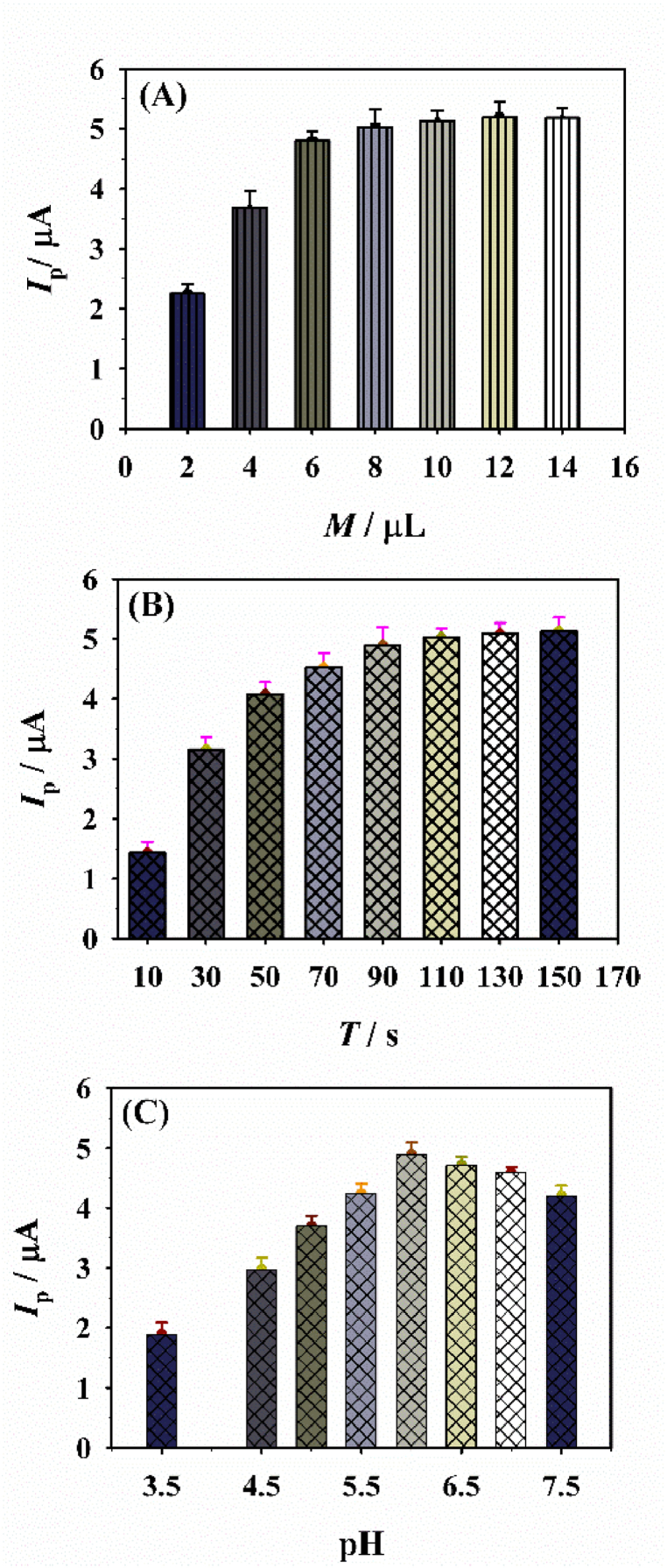
The influences from the (A) dropped amount of CNB-CD, (B) accumulation time and (C) pH value of PBS towards the DPV peak current of ATP at CNB-CD/GCE. Experiments parameters for A: accumulation time, 90 s; pH, 6.0; ATP concentration, 5.0 μM; potential range, 0.4–0.8 V. Experiments parameters for B: CNB-CD amount, 8 μL; pH, 6.0; ATP concentration, 5.0 μM, 0.4–0.8 V. Experiments parameters for C: CNB-CD amount, 8 μL; accumulation time, 75 s; ATP concentration, 5.0 μM, 0.4–0.8 V.

To obtain the best detection conditions of CNB-CD/GCE for ATP analysis, the optimization was performed by differential pulse voltammetry (DPV), Fig. 5 displays the influences from the used amount of the CNB-CD dispersion, the accumulation time towards ATP, and the pH value of PBS at CNB-CD/GCE. From Fig. 5A, it's found the DPV current of ATP molecules increases along with the increase of CNB-CD amount, the related maximum value presented at 8.0 μL of dispersion which exhibits best sensing ability of CNB-CD. Fig. 5B showed the DPV current increases also with the increase of accumulation time, and the maximum DPV current observed at 75 s. In addition, the influence from the pH value (PBS) was evaluated and the achieved results revealed that the best DPV response is at pH value of 6.0 (Fig. 5C). In conclusion, the optimized parameters are 8.0 μL for the CNB-CD dispersion, 75 s for the accumulation time and 6.0 pH for PBS.

**Fig. 6:**
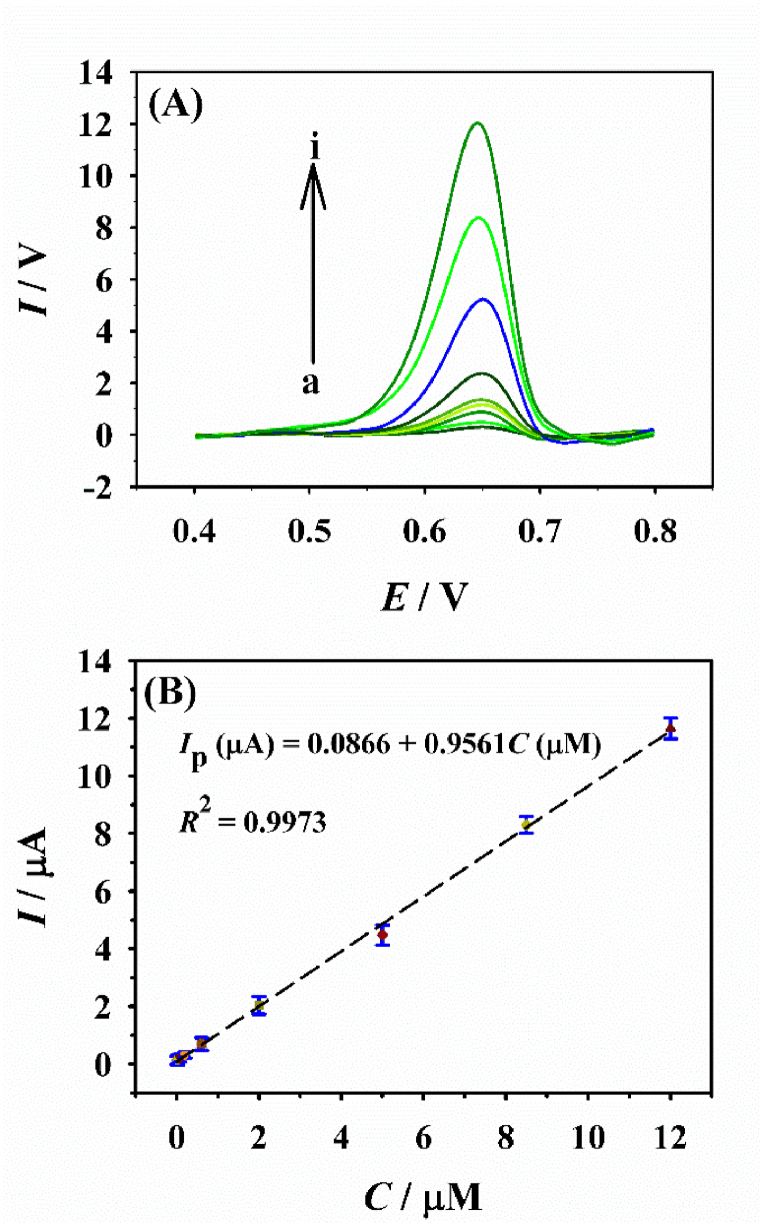
(A) The DPV responses of CNB-CD/GCE towards different ATP concentrations from a to i: 0.005, 0.02, 0.08, 0.2, 0.6, 2.0, 5.0, 8.5, and 12.0 μM. (B) The calibration plot for ATP detection.

Under the best parameters, the electrochemical analysis of ATP was performed by CNB-CD/GCE with DPV (Fig. 6). It was noted the peak currents at ∼0.65 V of ATP increase with the addition of the ATP degree (Fig. 6A). Via plotting the DPV currents against the ATP degrees, it's found from Fig. 6B that the signal values exhibited a wide linearity refer to the ATP degrees from 0.005 to 12.0 μM. The related equation is *I*_p_ (μA) = 0.0866 + 0.9561*C* (μM) (*R*^2^ = 0.9973), and the LOD value of ATP is 0.002 μM (S/N = 3). Table 1 displayed the comparisons between this work and previous works, it can be noted that the LOD value obtained in this work is lower and the linearity is larger than those presented by the most of previous works (Table 1); meanwhile, the preparation method of CNB-CD is very simple and the cost is considerable low.

**Table 1:** Comparison of different electrochemical sensors for the detection of ATP.

Sensors	Potential	pH	Method	Linear range [μM]	LOD [μM]	Reference
Carbon nanotube paste electrodes	0.73	7.0	DPV	0.01–1.5	0.0029	[3]
Hollow nanocarbon sphere/GCE	0.92	4.0	DPV	0.05–14.0	0.01	[46]
Ce–ZnO/GCE	0.7	7.0	DPV	0.01–500.0	0.0063	[11]
CoFe_2_O_4_/RG/ionic liquid/GCE	0.8	2.5	DPV	0.1–7.5	0.02	[14]
Graphene-carbon nanotube/GCE	0.65	6.0	DPV	0.7–10.0	0.07	[16]
Surfactant modified carbon nanotube paste electrode	0.6	7.0	CV	0.2–7.0	0.035	[47]
Biopolymers blend films/ITO	0.72	7.0	DPV	1.5–50.0	0.595	[48]
Carbon black/cooper nanoparticles/GCE	0.88	3.0	SWV	2.2–25.0	0.09	[49]
CNB-CD/GCE	0.65	6.0	DPV	0.005–12.0	0.002	This work

### 3.3 Selectivity, stability and repeatability

**Fig. 7:**
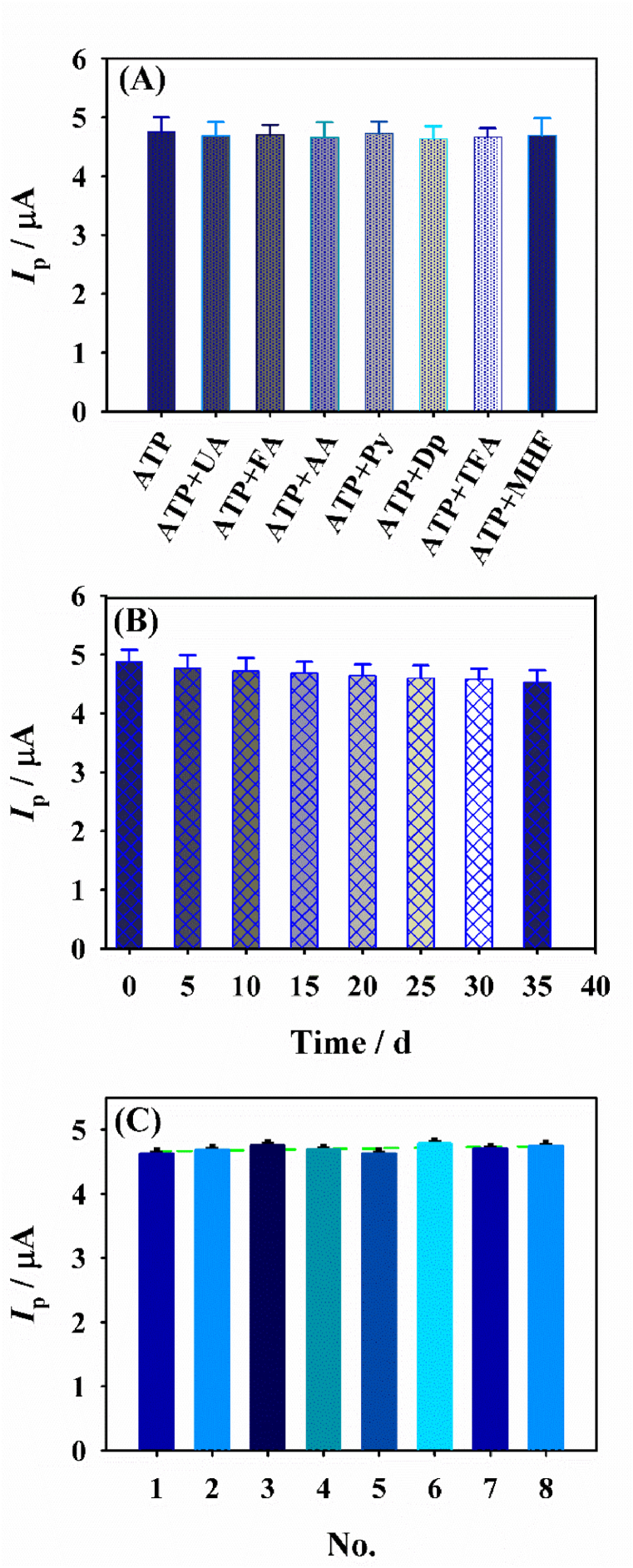
(A) The selectivity of response of eight different CNB-CD/GCE; (B) stability and reproducibility (C) of CNB-CD/GCE.

For revealing the selectivity of the developed sensor, the detection of ATP was measured in the presence of some interfering substances including 10 times of UA, FA, AA, Py, Dp, TFA and MHF. Interestingly, no interference can be observed (Fig. 7A), revealing CNB-CD/GCE has superior selectivity. Next, via recording the DPV signals of ATP for 35 days, the stability of the modified electrode was studied, the final results suggested no apparent reduction in the peak current appeared(<9.0 %) (Fig. 7B). In addition, for investigating the repeatability of the as-designed CNB-CD sensor, eight fabricated CNB-CD/GCE individually was adopted to detect 4.0 μM ATP, a low relative standard deviation value (<3.73 %) was observed for the peak currents (Fig. 7C).

## 4 Conclusions

In conclusion, through synthesizing CNB-CD nanohybrid as a novel electrode nanomaterial, a simple, highly sensitive and effective electrochemical sensor of ATP was developed successfully. On the basic of the collaborative superiorities from CNB nanoparticles and β-CD molecules, the as-constructed CNB-CD/GCE offers prominent analytical performances for ATP, which possesses a considerably low LOD (0.002 μM) and large linearity (0.005–12.0 μM). In addition, the repeatability and stability as well as the selectivity of CNB-CD/GCE is also desirable, it's thus expected that this work has important application for the electrochemical analysis of ATP and other targets.

## Data availability

Data will be made available on request.

## CRediT authorship contribution statement

**Jian Wang:** Writing – original draft, Validation, Software, Investigation, Formal analysis, Data curation, Conceptualization. **Xiuzhi Xu:** Validation, Formal analysis, Conceptualization. **Zhulai Li:** Writing – review & editing, Software, Investigation. **Bin Qiu:** Writing – review & editing, Supervision, Methodology, Funding acquisition, Conceptualization.

## Declaration of competing interest

The authors declare that they have no known competing financial interests or personal relationships that could have appeared to influence the work reported in this paper.
